# Characterization of the complete mitochondrial genome of the Tianjun yak (*Bos grunniens*)

**DOI:** 10.1080/23802359.2019.1692716

**Published:** 2019-11-20

**Authors:** Xian Guo, Xiaoyun Wu, Pengjia Bao, Suonan Zhao, Jinshou Ma, Shenbujia Yang, Min Chu, Xuezhi Ding, Xixi Yao, Chunnian Liang, Jie Pei, Ping Yan

**Affiliations:** a Key Laboratory of Yak Breeding Engineering of Gansu Province, Lanzhou Institute of Husbandry and Pharmaceutical Sciences, Chinese Academy of Agricultural Sciences, Lanzhou, People’s Republic of China;; b Institute of Animal Husbandry and Veterinary Medicine of Haibei Tibetan Autonomous Prefecture, Xihai, People’s Republic of China;; c Datong Cattle Farm in Qinghai Province, Xining, People’s Republic of China

**Keywords:** High-throughput sequencing, iterative mapping, mitochondrial gneome, phylogenetic analysis, yak breed

## Abstract

Tianjun yak (*Bos grunniens*) is a yak breed with strong adaptation to the high-elevation, cold and anoxic environments. Its complete mitochondrial genome is 16,323 bp long with an asymmetric base composition, and harbors the 37 typical mitochondrial genes and one noncoding control region. The PCGs are initiated with the typical ATA or ATG codons, and are terminated with TAA, TAG or the incomplete stop codon T––. Phylogenetic analysis suggests that Tianjun yak is most closely related to the polled yak.

Tianjun yak (*Bos grunniens*) is a local yak breed from Tianjun County, Haixi Mongolian-Tibetan Autonomous Prefecture, Qinghai Province, China, and has a strong adaptation to the high-altitude, cold and anoxic environments. To facilitate its genetic assays, its complete mitochondrial genome was assembled from high-throughput Illumina sequencing data in the present study. The annotated sequence has been deposited into GenBank under the accession number MN163006.

A blood sample of Tianjun yak was collected from Doukuer Village, Suli Township, Tianjun County (38°69′N, 98°04′E). A voucher specimen is held in the Key Laboratory of Yak Breeding Engineering of Gansu Province, Lanzhou Institute of Husbandry and Pharmaceutical Sciences (Lanzhou, Gansu Province, China). The genomic DNA coded as NO.20190824, which was extracted from Tianjun yak, is stored at −80 °C (ultra deep-freeze refrigerator) in the sample storage room of our department. Genomic DNA isolation were carried out with the QIAamp DNA Blood Mini Kit (Qiagen, Valencia, CA). Library construction and high-throughput sequencing with the Illumina HiSeq X™ Ten Sequencing System (Illumina, Valencia, CA) were conducted by Annoroad Gene Technology (Beijing, China). In all, 3.02 Gb of raw data were retrieved, and were used for mitochondrial genome assembly using MITObim v1.9 (Hahn et al. 2013) with a previously published sequence (JQ692071) (Qiu et al. 2012) as the initial reference. Mitogenome annotation was done by aligning against those of closely related taxa available from GenBank, and adjusted if necessary according to the prediction of the MITOS web server (Bernt et al. 2013; Al Arab et al. 2017).

The mitochondrial genome of Tianjun yak is structurally similar to those of previously published ones of *Bos grunniens* (e.g. Wu et al. 2016, 2019). It is 16,323 bp long with an asymmetric base composition (33.7%A, 25.8%C, 13.2%G, and 27.3%T), and encodes the 37 typical animal mitochondrial genes (13 protein-coding genes/PCGs, 22 tRNAs and two rRNAs). The PCGs are initiated with either ATA (*ND2, ND3,* and *ND5*) or ATG (the 10 others) codon. Three types of stop codons are employed, i.e. TAG (*ND2*), TAA (*ATP6*, *ATP8*, *COX1*, *COX2*, *CYTB*, *ND1*, *ND4L*, *ND5,* and *ND6*) and the incomplete stop codon T (*COX3, ND3,* and *ND4*). The 22 tRNAs range in size from 60 (*tRNA-Ser^AGN^*) to 75 bp (*tRNA-Leu^UUR^*). The two rRNAs are 957 bp (*12S rRNA*) and 1571 bp (*16S rRNA*) in size, respectively. The control region is 893 bp long, and is located between *tRNA-Pro* and *tRNA-Phe*.

A Bayesian phylogenetic tree was reconstructed using MrBayes v3.1.1 (Ronquist and Huelsenbeck 2003) as implemented in the program TOPALi v2.5 (Milne et al. 2009) to investigate its relationship with 12 other yak breeds with available mitochondrial genomes (Figure 1). “HKY + I” was selected as the best-fit nucleotide substitution model according to the Bayesian Information Criterion (BIC). All 13 PCGs were concatenated into a single “supergene” after the removal of stop codons, and were then used for the phylogenetic analysis. The outgroup taxa included are two species within the genus *Bison*: *Bison bison* (GU946976) (Douglas et al. 2011) and *Bison priscus* (KX269111) (Froese et al. 2017). It suggests that Tianjun yak is more closely related to the polled yak (KM658599) (Chu et al. 2016) than to the other 11 yak breeds.

**Figure 1.**
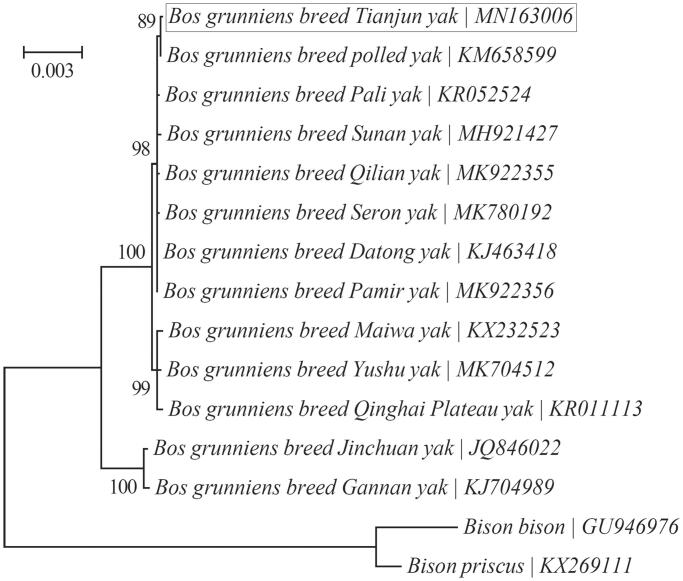
Phylogenetic relationships of 13 yak breeds based on the Bayesian analysis of the concatenated sequences of 13 mitochondrial protein-coding genes (alignment size: 10,370 bp). The support values are shown next to the nodes. Two *Bison* species were included as outgroup taxa.
